# A Micelle Electrolyte Enabled by Fluorinated Ether Additives for Polysulfide Suppression and Li Metal Stabilization in Li-S Battery

**DOI:** 10.3389/fchem.2020.00484

**Published:** 2020-06-19

**Authors:** Yangzhi Zhao, Chen Fang, Guangzhao Zhang, Dion Hubble, Asritha Nallapaneni, Chenhui Zhu, Zhuowen Zhao, Zhimeng Liu, Jonathan Lau, Yanbao Fu, Gao Liu

**Affiliations:** ^1^Lawrence Berkeley National Laboratory, Energy Storage and Distributed Resources Division, Berkeley, CA, United States; ^2^Advanced Light Source, Lawrence Berkeley National Laboratory, Berkeley, CA, United States; ^3^Department of Polymer Engineering, University of Akron, Akron, OH, United States; ^4^Department of Chemical Engineering and Materials Science, Michigan State University, East Lansing, MI, United States

**Keywords:** polysulfide suppression, Li metal stabilization, fluorinated ether electrolyte, micelle-like complex formation, solvation mechanism, high coulombic efficiency

## Abstract

The Li-S battery is a promising next-generation technology due to its high theoretical energy density (2600 Wh kg^−1^) and low active material cost. However, poor cycling stability and coulombic efficiency caused by polysulfide dissolution have proven to be major obstacles for a practical Li-S battery implementation. In this work, we develop a novel strategy to suppress polysulfide dissolution using hydrofluoroethers (HFEs) with bi-functional, amphiphlic surfactant-like design: a polar lithiophilic “head” attached to a fluorinated lithiophobic “tail.” A unique solvation mechanism is proposed for these solvents whereby dissociated lithium ions are readily coordinated with lithiophilic “head” to induce self-assembly into micelle-like complex structures. Complex formation is verified experimentally by changing the additive structure and concentration using small angle X-ray scattering (SAXS). These HFE-based electrolytes are found to prevent polysulfide dissolution and to have excellent chemical compatibility with lithium metal: Li||Cu stripping/plating tests reveal high coulombic efficiency (>99.5%), modest polarization, and smooth surface morphology of the uniformly deposited lithium. Li-S cells are demonstrated with 1395 mAh g^−1^ initial capacity and 71.9% retention over 100 cycles at >99.5% efficiency—evidence that the micelle structure of the amphiphilic additives in HFEs can prohibit polysulfide dissolution while enabling facile Li^+^ transport and anode passivation.

## Introduction

Over the past two decades, tremendous effort has been invested in developing clean and renewable energy technologies that may address urgent environmental concerns surrounding fossil fuels. As of 2018, renewable sources accounted for >10% of energy consumption in the United States. Two major technologies, solar and wind, have broken through technical barriers and currently hold a dominant share in the global market of renewables; however, because of their intermittent nature, energy storage must be integrated across multiple scales, from power grid to personal device, to allow increasing deployment of these resources. This is particularly important in the case of electric vehicles, which demand both high power and energy density to support running a powertrain over long distances. Li-ion batteries have proven to be an enabling technology in this regard, and their capacity has greatly improved since their commercial debut in 1991. Nevertheless, state-of-the-art Li-ion performance has begun to bottleneck as energy density approaches the theoretical limit of classic intercalation chemistry (~400 Wh kg^−1^). (Fan et al., 2018) In order to satisfy global demand for high-capacity energy storage, new chemistry and electrode materials have been proposed such as the lithium-sulfur (Li-S) battery (Song et al., 2013a; Yin et al., 2013; Hietala et al., 2018; Fang et al., 2019). This redox couple has a theoretical energy density of 2600 Wh kg^−1^, and material costs are expected to be low due to the worldwide abundance of sulfur, which makes Li-S stand out as a promising next-generation storage solution. However, practical implementation of this technology has been delayed by fundamental challenges such as lithium polysulfide (Li_2_S_n_, *n* > 4) dissolution and redox shuttling, low conductivity of sulfur/sulfide materials, detrimental corrosion and dendrite formation on lithium anode etc (Ma et al., 2014; Li et al., 2017; Fang et al., 2019). Among these, polysulfide dissolution is often considered the most significant complication, as it is responsible for the poor cycling stability and low coulombic efficiency usually observed in Li-S batteries. At present, many of the strategies reported to address this issue are focused on modification of electrode structures. These strategies include physically and/or chemically confining polysulfide intermediates within a hierarchical matrix (Wang et al., 2015; Wu et al., 2015) or crosslinked organic structure (Li et al., 2019), blocking contact between polysulfide and electrolyte by building protective layers/shells on the cathode surface (Hu et al., 2016; Wu et al., 2018), forming stable/protective SEI layers on the anode surface using electrolyte additives (e.g., LiNO_3_) (Aurbach et al., 2009), and developing template configurations that allow solid-state conversion of nanoconfined sulfur (S_2−4_) (Xin et al., 2012; Li et al., 2014).

Alternatively, a more effective solution to this problem can often be derived from electrolyte engineering, which requires understanding the interactions between polysulfides and electrolyte species. Typically, Li-S batteries utilize an electrolyte of 1.0M lithium bis(trifluoromethanesulfonyl)imide (LiTFSI) dissolved in a 50:50 v/v solvent mixture of 1,2-dimethoxyethane (DME) and 1,3-dioxolane (DOL). Polysulfide dissolution usually occurs at the early stages of discharge when sulfur is converted to long-chain lithium polysulfides (e.g., Li_2_S_6_), which subsequently dissolve due to the favorable coordination of Li^+^ with electron-donating sites on the solvent (Park et al., 2013a; Vijayakumar et al., 2014). Therefore, seeking an alternative electrolyte solvent with weaker donating capability, yet good chemical and electrochemical stability, may provide an answer to this problem. Also, polysulfide solubility may be reduced with an increase in lithium salt concentration as per the so-called common ion effect; (Vijayakumar et al., 2014) intuitively, one might imagine that pre-existing solvated Li^+^ ions “use up” available coordination sites, leaving fewer remaining to interact with lithium polysulfides. As a result, high-concentration electrolytes i.e., room-temperature ionic liquids, (Park et al., 2013a,b; Song et al., 2013b) solvent-in-salt, (Suo et al., 2013), and solvate ionic liquids (Dokko et al., 2013; Ueno et al., 2013; Zhang et al., 2016) have all proven quite effective at discouraging polysulfide dissolution, despite their high viscosity and subsequent poor lithium transport properties. More recently, several researchers have addressed this problem through dilution of concentrated electrolytes with poorly-coordinating solvents; work reported by Weller et al. demonstrated a low-density electrolyte with high lithium salt concentration to suppress polysulfide dissolution using hexyl methyl ether in combination with DOL (Weller et al., 2019). The Wang group has recently reported good Li-S cell performance achieved from developing a “localized high concentration electrolyte” using a fluoroalkyl ether additive blended with DME electrolyte (Zheng et al., 2019). Of special interest are hydrofluoroethers (HFEs), a class of solvents with moderate polarity but low solvation strength for lithium. Moreover, lithium anode corrosion can be significantly reduced in the electrolyte with the presence of chemically inert HFEs, leading to high coulombic efficiency (Fan et al., 2018). Additional benefits include improved cell safety and expanded electrochemical window, ascribed to the reduced flammability and good electrochemical stability of these solvents. One particular HFE commonly reported for Li-S application is 1,1,2,2-tetrafluoroethyl-2,2,3,3-tetrafluoropropyl ether (TTE); (Dokko et al., 2013; Cuisinier et al., 2014; Azimi et al., 2015; Lu et al., 2016; See et al., 2016; Lee et al., 2017) nevertheless, since HFEs like TTE can only barely dissolve lithium salts e.g., LiTFSI on their own, (Ren et al., 2018) highly-lithium ion coordinating co-solvents are always necessary. In order to fully realize the advantages of HFEs, it is desirable to minimize polysulfide access to coordinating sites while still maximizing free Li^+^ content in the electrolyte.

Herein, we have designed two new, highly-fluorinated electrolyte solvents in order to suppress polysulfide dissolution while retaining good lithium salt concentration. Taking inspiration from ampiphilic surfactants, these HFE molecules were designed with a bi-functional structure, comprising one lithiophilic section and one lithiophobic section. An electrolyte was made by dissolving LiTFSI or LiFSI in HFE solvent at various concentrations, followed by dilution with inert TTE. We hypothesize that Li salt dissolution in this system follows a special solvation mechanism, resulting in formation of micelle-like complexes; these formed structures are essential to achieving simultaneous high solubility of lithium imide salts and low solubility of lithium polysulfides. Small angle X-ray scattering is used to provide experimental evidence of complex micelle formation. Li-S cells were fabricated by using HFE/TTE electrolyte, and galvanostatic charging/discharging was performed to study cycling stability and coulombic efficiency. Li||Cu cells were also fabricated for stripping/plating tests. The as-deposited lithium was imaged by scanning electron microscopy (SEM) to investigate the compatibility of HFE/TTE electrolyte with lithium metal anodes.

## Experiments

### Chemicals

2,2,3,3,4,4,4-Heptafluoro-1-butanol (TCI America), 1H,1H,2H,2H-Perfluorohexan-1-ol (Oakwood Products, Inc.), 1H,1H,2H,2H-Perfluoro-1-decanol (Oakwood Products, Inc.), 2-Methoxyethanol (Aldrich), Potassium hydroxide (Aldrich), Triethylamine (Aldrich), Dichloromethane (VWR), 1-Methyl-2-pyrrolidinone (NMP, anhydrous, Aldrich), poly(vinylidene fluoride) (PVDF, Mw = ~534000, Aldrich), sulfur (Aldrich), Ketjen black carbon (EC-600JD, Akzo Noble Functional Chemicals LLC), Denka black (Denka Co.), polypropylene separator (Celgard), lithium foil (Albemarle), lithium bis(trifluoromethane sulfonyl)imide (LiTFSI, 98+%, Alfa Aesar), lithium bis(fluorosulfonyl)imide (LiFSI) (98.0+%, TCI America), and baseline electrolyte 1.0M LiTFSI in DME/DOL (v:v=1:1) (UN2924, Gotion) were all used as received without further purification. 1,1,2,2-Tetrafluoroethyl 2,2,3,3-tetrafluoropropyl ether (TTE, >95.0%, TCI America) was dried over molecular sieves (4Å, Aldrich) to remove trace amount of moisture.

### Instrumentation

Copper foil with deposited lithium was recovered from disassembled coin cells (CR2032, MTI Corp.) in an Ar-filled glovebox after a lithium plating cycle, then carbon-taped to a specimen stub for SEM imaging using a JEOL JSM-7500F with an accelerating voltage of 15kV. Nuclear magnetic resonance (NMR) measurements were carried out on a Bruker Advance II 500 MHz NMR Spectrometer. NMR samples were prepared by mixing 20μL of the sample solution in 0.5 mL of chloroform-d (CDCl_3_). The mixture solution was then loaded in a quartz sample tube for measurement. Conductivity measurement was performed by using a conductivity meter (Seven2Go™ pro, METTLER TOLEDO) in the glovebox. For an accurate measurement, the probe tip has to be fully submerged in the electrolyte which requires about 5 ml electrolyte solution. The probe tip was cleaned by rinsing with the dilution solvent TTE and dried by Kimwipe between measurements. Transmission small angle X-ray (SAXS) scattering measurements were performed at Advanced Light Source (beamline 7.3.3), Lawrence Berkeley National Laboratory (LBNL) to analyze the size of the formed complexes. The *q* range is between 6 × 10^−4^ Å^−1^ and 0.3 Å^−1^; here q is wave-vector and *q* = 4π/λ sin (θ), λ is the wavelength and θ is the scattering angle. The X-ray energy was 10 keV (λ = 0.124 nm). Silver behenate (AgC_22_H_43_O_2_) was used for calibrating sample-detector distance and beam center. Electrolyte samples were first prepared and then sealed in quartz capillary tubes in an Ar-filled glove box prior to SAXS measurements due to the air-sensitive nature of the electrolytes. Solvent background was subtracted prior to the analysis. Subsequent data reduction and size distribution analysis were performed following standard procedure through Nika and Irena packages embedded in Igor software, respectively (Ilavsky and Jemian, 2009; Ilavsky, 2012). The 2D data is first converted to a 1D plot in the format of Intensity (I) vs. wave-vector (q) for size analysis. Model fitting and size distribution analysis were finally performed by using the Modeling tool from Irena package. The math equations used in the fitting model can be found in the supporting document.

### Electrode Fabrication and Electrochemical Measurements

In order to achieve good electrochemical performance, sulfur was first confined in a carbon matrix to form S/C composite. Sulfur and Ketjen black carbon were mixed in 7:3 weight ratio by mechanical ballmixing (CryoMill, Retsch) for 15 min. The mixture was sealed in a Teflon container and then annealed at 155 °C for 15 h in a tube furnace (Lindberg Blue M, Thermo Scientific). For slurry making, S/C composite, Denka black and premade PVDF binder solution (10 wt% in NMP) with addition to extra NMP solvent were mixed by mortar and pestle in a 6:3:1 mass ratio. The slurry was then doctor-bladed on an aluminum foil (20μm, All Foils Inc.) current collector and solvent allowed to evaporate at 40°C overnight. Cathode disk (dia. 12.7 mm) were punched from the electrode film and were further dried under vacuum for 12 h before transferring into an Argon-filled glovebox (Vacuum Technology Inc., <0.1ppm, H_2_O, and O_2_). Each cathode disc contained ~1.0 mg/cm^2^ mass loading of sulfur. The sulfur cathode disk and lithium foil anodes were assembled into CR2032 coin cell cases in the glovebox. Celgard 2400 polypropylene film was used as the cell separator. The assembled cells were placed in an isothermal chamber (30 °C) connected to a Maccor series 4000 cell tester for electrochemical performance measurement. Galvanostatic charging and discharging was performed between 1.4 and 2.7 V at a rate of C/10 (1C = 1672 mA/g). Similarly, Li||Cu coin cells were assembled for Li plating/stripping tests using 0.5M LiFSI in HFE as the electrolyte. To complete the test, Li film was first deposited on copper foil (15 μm, Fukuda Metal Foil and Power Co. Ltd) at a constant current and was then fully stripped to 1.0V, followed by repeating cycles of the same procedure. Capacity value is normalized based on the mass of sulfur in each electrode.

## Results and Discussion

### Micelle Formation in the Electrolytes

**Figure 1:**
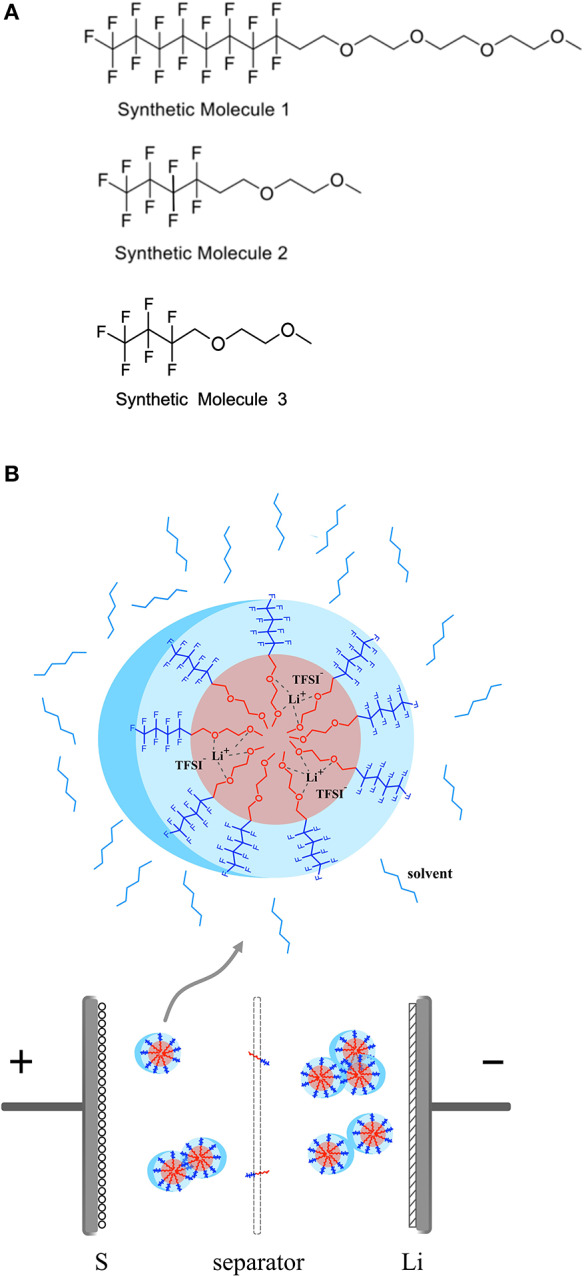
**(A)** Molecular structure of fluorocarbon ethylene oxides with different chain length denotated as F_8_EO_4_, F_4_EO_2_, and F_3_EO_1_ respectively. **(B)** Schematic diagram to show solvation mechanism of LiTFSI with HFE in the electrolyte and formation of micelle complex structure.

Our HFE molecules are designed to have a bi-functional, amphiphilic structure consisting of a fluorocarbon moiety and an ethylene oxide (EO) moiety on each end, respectively. The molecular structures of the class of synthesized HFE molecules—denoted as F_8_EO_4_, F_4_EO_2_, and F_3_EO_1_—are shown in Figure 1A. These molecules are synthesized following modified approaches from literature. The detailed synthesis procedure and (Fan et al., 2018) H NMR chemical shifts can be found in the supporting information (Zaggia et al., 2010). Both molecules possess the designed bi-functional structure but with different chain lengths. This unique “Janus” structure leads to existence of directly opposite properties within one molecule in which an electrolyte salt may dissolve following a special solvation mechanism, as depicted in Figure 1B. The EO moiety is lithiophilic and incompatible with fluorinated solvents, therefore able to coordinate with Li^+^, while the fluorocarbon moiety is lithiophobic but fluorophilic, which is more likely to associated with fluorinated solvents like TTE. It is hypothesized that this will result in the formation of micelle-like molecular complexes containing varying numbers of HFE molecules and salt ions. We expect such a solvation mechanism will suppress polysulfide dissolution and redox shuttling as the limited lithium coordinating ether groups are preoccupied with existing Li^+^ salt and not available for polysulfides, such that the solubility of polysulfides in the electrolyte is reduced. Similar to the kinetics theory of amphiphilic block co-polymer, the formation of micelles in our electrolyte is a self-assembly process which is mainly driven by the incompatibility of EO groups with fluorinated solvent TTE, and is counteracted with repulsion and unfavorable configuration of joined molecules, synergistically creating an equilibrium of nanostructures. Such equilibrium can be affected by disturbing variables such as temperature, pressure, PH value, and salt addition etc., causing redistribution of joined molecules, fusion/fission of micelles, and shape change of micelle structures (Lund et al., 2013). Hence, spherical, cylindrical or lamellar of micelles are commonly existed, depending on the concentration of the amphiphilic HFE and the lithium salt in the TTE solvent. By virtue of lithiophilic attraction, Li^+^ dissociated from the salt will spontaneously assemble around micelle core area, forming special channels for ion conduction. In addition, micelles are mobile in the electrolyte, Li^+^ diffusion paths are likely shortened by those channels and ion conduction in the electrolyte is thus facilitated.

**Figure 2:**
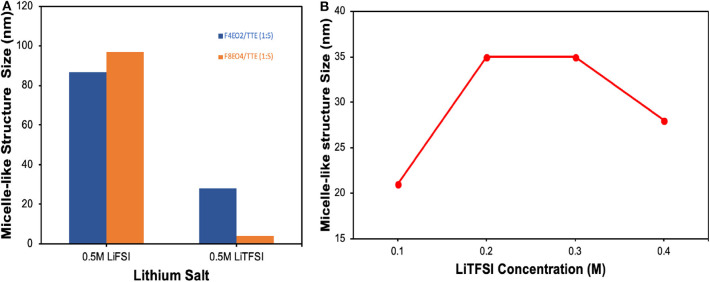
Size information extracted from SAXS measurement for **(A)** 0.5M salts dissolved in HFE/TTE 1:5 (v/v) as a function of salt and HFE additive identity. **(B)** Complex aggregate size as a function of LiTFSI salt concentration in F_4_EO_2_/TTE 1:5 (v/v).

It is important to provide experimental evidence for this special solvation mechanism and formation of the expected micelle-like complex structure. Small angle X-ray scattering (SAXS) is based on Rayleigh scattering and often used to identify particle size and distribution for colloidal systems by probing response in electron density contrast of molecules or aggregates dispersed in solution (Borchert et al., 2005; Pabisch et al., 2015; Zhao et al., 2020). Figure 2 summarizes size information extracted from SAXS measurement for actual electrolyte solutions comprised of LiTFSI and LiFSI salts dissolved in a mixture of HFE additive and TTE solvent. Figure S1 displays the 1D plot and Modeling fitting process performed through Nika and Irena packages from Igor Pro software. All results are plotted after the background of HFE and TTE solvent was subtracted. As SAXS detection limit typically falls in the particle range of 2–100 nm, where dust impurity signals are screened, the obtained size information thus primarily represents structures formed between molecules of lithium salts and HFE. Figure 2A displays complex aggregate size as a function of salt and HFE identity. It can be clearly seen that salt identity plays a role in determining dimensions of the complex aggregates—much larger size is observed for LiFSI. This could be attributed to the participation of more LiFSI units in the formation of one single complex under the same salt concentration due to easier incorporation of the smaller anions. In addition, charge repulsion might play a role in limiting aggregate size, which would indicate that LiFSI-based aggregates are larger due to more extensive ion pairing. Interestingly, it seems that the smaller HFE molecule (F_4_EO_2_) tends to form larger aggregates with the larger LiTFSI salt, while larger HFE molecule (F_8_EO_4_) prefers LiFSI. Prior report of solvation behavior between these salts in bulk fluorinated media supports our observation in micelles, which would be a fruitful area for further investigation (Shah et al., 2017). Figure 2B reflects complex aggregate size change with LiTFSI salt concentration. After an initial rise, the average size levels off in the range of 0.2–0.3M before decreasing. This makes sense in that complexes will continue to grow with concentration until a saturation point is reached. After 0.3M, “over saturation” occurs for the micelle-like geometry and a more-complex structure e.g., lamellae is likely to form at this point; thus, the decreasing size might reflect an inter-lamellar distance rather than the size of isolated complexes. In summary, SAXS provides straightforward and solid experimental evidence to support the existence of complexes formed in the electrolyte at different stages, validating our proposed solvation mechanism. More insights into complex formation, including the dynamic evolution of coordination between molecules and ions, can be further demonstrated by molecular dynamics (MD) simulation which is beyond the scope of this work.

### Li-S Cell Performance

Prior to cell fabrication and cycling test, the solubility of lithium salts LiTFSI and LiFSI in our highly-fluorinated HFE additives were tested. Results showed that maximum solubility reached 3.5M–4.0M for both salts in HFE solvents; however, the solutions at that concentration were too viscous for cell application. Therefore, the highly concentrated solutions were diluted with chemically-inert TTE. Literature suggests that optimum ionic conductivity usually falls in the range of 0.5–1.0M for lithium electrolytes (Walls et al., 2003). Table 1 lists the measured ionic conductivity of 0.5M LiTFSI dissolved in HFEs with varying TTE dilution ratios. All electrolytes in the table reveal a conductivity at the level of 10^−4^-10^−3^ S/cm, which is highly comparable with commercial organic liquid electrolyte. Higher ionic conductivity is achieved with the smallest HFE molecule F_3_EO_1_. This is probably due to the formation of larger complexes which reduce overall ion diffusion path and facilitates ion conduction. Another interesting finding is higher conductivity is favored by the 1:1 dilution ratio because more ion conducting EO groups are present in the electrolyte. Taking into account ionic conductivity and the desired wettability associated with appropriate viscosity, 0.5M concentration was selected for further study, including Li-S cell fabrication.

**Table 1:** Room-temperature (25°C) ionic conductivity (σ) for 0.5M LiTFSI electrolyte with various HFE additives (F_3_EO_1_ and F_8_EO_4_) and TTE dilution ratios.

**HFE additive : TTE by volume**	**σ (mS/cm)**	
	**F_**3**_EO_**1**_**	**F_**8**_EO_**4**_**
1:1	3.2	0.61
1:5	1.05	0.35

**Figure 3:**
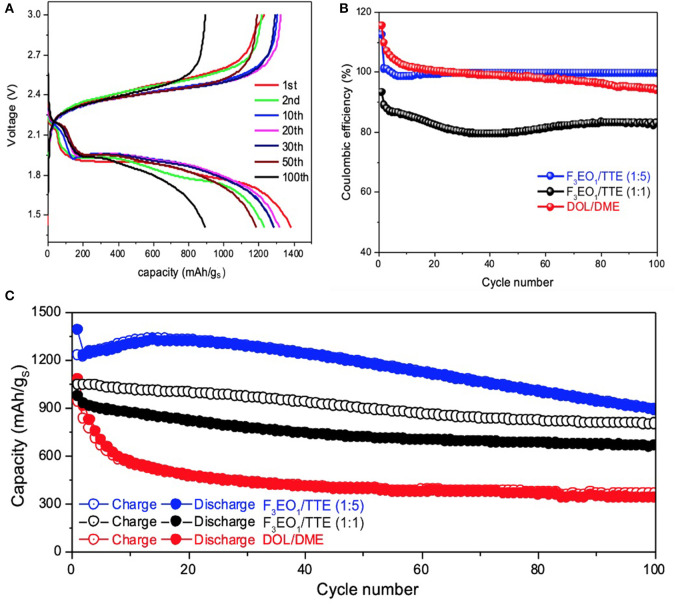
Electrochemical performance of Li-S cells with 0.5M LiTFSI in F_3_EO_1_/TTE electrolyte: **(A)** voltage profiles for 1:5 F_3_EO_1_/TTE volume ratio; **(B)** coulombic efficiency comparison of various electrolytes; **(C)** cycling stability comparison of various electrolytes.

Electrochemical performance of electrolytes 0.5M LiTFSI in F_3_EO_1_/TTE and 0.5M LiTFSI in F_8_EO_4_/TTE in comparison with baseline DOL/DME electrolyte are displayed in Figure 3 and Figure S2, respectively. Voltage curves with similar features are observed for both HFE additives. In contrast to the baseline electrolyte, the characteristic first discharge plateau does not begin until below 2.3V and comprises a lower percentage of the overall capacity, suggesting that the formation of long chain polysulfides (Li_2_S_n_, n≥6) is disincentivized in the HFE additive electrolyte. However, over extended cycling the second discharge plateau both attenuates in capacity and gradually sinks to lower voltage; this behavior is quite common in sparingly-solvating electrolytes and indicates sluggish kinetics due to the increasingly solid-state nature of conversion. Because polysulfides barely dissolve, sulfur on cathode surfaces will go through solid-solid phase transformations rather than solid-liquid and vice-versa, as is typical; however, tailored electrode design can help to mitigate polarization in this case (Fan and Chiang, 2017; Helen et al., 2019; Seita et al., 2019).

Figures 3B,C compares coulombic efficiency (CE) and cycling stability of various electrolytes. As the Li-S cells start from discharge cycle (sulfur lithiation), the Li_2_S_x_ electrode usually cannot be fully delithiated on the following charge cycle, causing the initial coulombic efficiency beyond 100% for both baseline electrolyte and F_3_EO_1_/TTE (1:5) electrolyte.

The CE remains constant around 99.5% for F_3_EO_1_/TTE (1:5) electrolytes up to 100 cycles, while it decays rapidly for baseline electrolyte and falls below CE line of F_3_EO_1_/TTE (1:5) electrolyte after only 30 cycles. The HFE additive electrolytes also shows superior cycling performance (Figure 3C), outperforming the baseline in both initial capacity (1395 vs. 1070 mAh/g_S_) and retention over 100 cycles (71.9 vs. 40.2%). Again, F_3_EO_1_/TTE (1:5) electrolyte exhibits optimal performance due to its combination of appropriate ionic conductivity with poor polysulfide solvation, while the baseline electrolyte exhibits decay as a result of polysulfide dissolution and shuttling. Similar but slightly inferior electrochemical performance is observed for the F_8_EO_4_/TTE (1:5) counterpart shown in Figures S2B,C, which can likely be blamed on poorer lithium transport. The overall electrochemical performance of F_3_EO_1_/TTE (1:1) electrolyte, however, is worse than 1:5 formulation, exhibiting both lower capacity and significantly worse CE, despite its better ionic conductivity. This highlights the importance of striking an appropriate balance between lithiophilic and lithiophobic content in the electrolyte, as the 1:1 electrolyte appears to have enough uncoordinated EO functionality to support substantial polysulfide shuttling. Generally speaking, we find that the use of designed HFE additives can significantly improve the coulombic efficiency and cycling stability of Li-S cells thanks to simultaneous achievement of good lithium transport and suppression of polysulfide solubility. Despite the larger polarization induced by a solid-state conversion mechanism, the high observed capacity and CE suggest a promising future for designed HFEs in Li-S battery application. The inherent trade-off between charge transfer kinetics and cycling performance may be addressed by further refinement of the electrode architecture on a case-by-case basis.

### Li Metal Plating/Stripping CE and Cycling Stability

**Figure 4:**
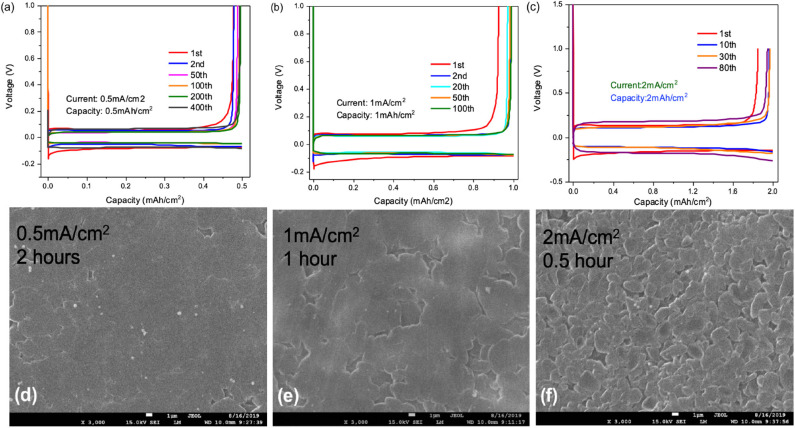
Voltage profiles for Li plating/stripping test by using 0.5M LiFSI in F_3_EO_1_/TTE (1:5) electrolyte under various conditions: **(a)** 0.5 mA/cm^2^ current density and 0.5 mAh/cm^2^ deposition capacity; **(b)** 1.0 mA/cm^2^ current density and 1.0 mAh/cm^2^ deposition capacity **(c)** 2.0 mA/cm^2^ current density and 2.0 mAh/cm^2^ deposition capacity; **(d–f)** SEM images (3000×, magnification) of deposited surfaces at various current density and constant 1.0 mAh/cm^2^ deposition capacity.

Li||Cu cells were employed to study the cycling stability and coulombic efficiency of the designed F_3_EO_1_ additive electrolyte in regards to anode performance. As previously discussed, HFEs are hypothesized to have better chemical compatibility with lithium metal due to their rapid breakdown into passivating LiF (Zhang et al., 2017); thus, high CE and cycling stability are expected over long term cycling. First, the effect of various current densities were investigated to study rate capability of the cell. As displayed in Figure 4a, at 0.5 mA/cm^2^, initial CE was only about 90% but continuously increased with stripping/plating cycles after the Cu surface became passivated. Notably, >99.5% CE was observed after 200 cycles (Figure S3) and likewise the polarization decreases with cycling to 35 mV because of the increasing surface area (Wu et al., 2017), indicating that the HFE/TTE electrolyte is highly stable with lithium deposition. Similar behavior was observed at 1.0 mA/cm^2^ current density (Figure 4b); other than the expected slight increase in overpotential, CE also reaches >99.5% after only 100 cycles. Figure 4c shows that CE is only slightly compromised at 2.0 mA/cm^2^ current rate. This comparison demonstrates the good rate capability of Li metal anodes in HFE solvent electrolyte.

SEM images of deposition morphology are shown in Figures 4d–f following 1.0 mAh/cm^2^ deposition at varying current density. Very smooth lithium surfaces were observed at 0.5 and 1.0 mA/cm^2^ rate. A slightly rougher surface with lithium nodule formation appears at 2.0 mA/cm^2^ rate; however, no dendritic growth was found on these surfaces indicating a good suppression of dendrite formation using this HFE additive electrolyte.

## Conclusion

We have reported a novel strategy to suppress polysulfide dissolution in Li-S cells utilizing an HFE with bi-functional and amphiphilic structure (one lithiophilic section and one lithiophobic section) similar to that of a surfactant. The electrolyte used in this work was made by dissolving LiTFSI or LiFSI in HFE additive solvent at various concentrations with dilution of 1,1,2,2-tetrafluoroethyl-2,2,3,3-tetrafluoropropyl ether (TTE) solvent. We have found that lithium salt dissolution follows a special solvation mechanism whereby dissociated lithium ions readily coordinate with limited donating groups in the HFE to form micelle structured complexes, which was experimentally verified by small angle X-ray scattering (SAXS). According to solubility tests, LiTFSI and LiFSI render the maximum concentration of ~4.0 M in these liquids; dilution to 0.5 M in HFE/TTE 1:5 (v/v) produces ionic conductivities similar to commercial organic electrolytes. Superior cycling stability and higher coulombic efficiency was observed for Li-S cells fabricated with an HFE/TTE electrolyte compared to those using benchmark DME/DOL electrolyte. A high coulombic efficiency for lithium metal plating/stripping together with modest polarization in Li||Cu cells indicates good chemical compatibility of HFE with lithium metal. In addition, dendrite formation was greatly suppressed as only smooth surface morphology was observed for the deposited lithium. Future work will include demonstration of the dynamic evolution of coordination between solvent molecules and lithium salts by molecular dynamics (MD) simulation, and optimization of battery performance by integrating electrode adaptation strategies.

## Data Availability Statement

The raw data supporting the conclusions of this article will be made available by the authors, without undue reservation, to any qualified researcher.

## Author Contributions

YZ performed electrochemical testing and drafted the paper. CF synthesized materials. GZ synthesized materials and performed electrochemical testing. AN and CZ conducted SAXS measurement and analyzed data. ZZ drafted Figure 1. ZL, JL, and YF participated in discussion and reviewed the paper. GL conceived the experiments, analyzed data, and supervised the research. All authors contributed to writing the paper.

## Conflict of Interest

The authors declare that the research was conducted in the absence of any commercial or financial relationships that could be construed as a potential conflict of interest.
