# Supplementary material for: A Micelle Electrolyte Enabled by Fluorinated Ether Additives for Polysulfide Suppression and Li Metal Stabilization in Li-S Battery
Source: Front Chem. 2020 Jun 19;8:484. doi: 10.3389/fchem.2020.00484 (PMC7317089; doi:10.3389/fchem.2020.00484)
Supplement: Supplementary file 1 [file Presentation_1.pdf]

Supporting Information

A Micelle Electrolyte Enabled by Fluorinated Ether Additives for Polysulfide Suppression and Li Metal Stabilization in Li-S Battery

Yangzhi Zhao[1], Chen Fang[1], Guangzhao Zhang[1], Dion Hubble[1], Asritha Nallapaneni[2,3], Chenhui Zhu[2], Zhuowen Zhao[4], Zhimeng Liu[1], Jonathan Lau[1], Yanbao Fu[1], and Gao Liu[1]*

1. Energy Storage and Distributed Resources Division, Lawrence Berkeley National Laboratory, 1 Cyclotron Road, Berkeley, CA 94720, United States
2. Advanced Light Source, Lawrence Berkeley National Laboratory, 1 Cyclotron Road, Berkeley, CA 94720, United States
3. Department of Polymer Engineering, University of Akron, Akron, OH 44325 United States
4. Department of Chemical Engineering and Materials Science, Michigan State University, East Lansing, MI 48824, United States

* Corresponding author

Email address: gliu@lbl.gov

1. Materials Synthesis

Molecule **1** (F_8EO_4), 14,14,15,15,16,16,17,17,18,18,19,19,20,20,21,21,21-heptadecafluoro-2,5,8,11-tetraoxahenicosane.

Molecule **2** (F_4EO_2), 1,1,1,2,2,3,3,4,4-nonafluoro-6-(2-methoxyethoxy)hexane

Molecule **3** (F_3EO_1), 1,1,1,2,2,3,3-heptafluoro-4-(2-methoxyethoxy)butane

General procedure for synthesis of electrolyte molecules **1-3**: 90 mmol of either 2-methoxyethanol or triethylene glycol methyl ether was mixed with 94.5 mmol 4-toluenesulfonyl chloride and 180 mmol triethylamine in 270 ml dichloromethane at 0 °C. The mixture was stirred overnight, extracted with dichloromethane, and washed with dilute HCl, saturated $NaHCO_3$, and water. The tosylate products were concentrated under reduced pressure. The crude products were used without further purification. 76 mmol tosylate and 38 mmol fluorinated alcohol were mixed in 23 ml 45wt% KOH aqueous solution and 23 ml NMP. The mixture was stirred at 50 °C for 5 hours and 70 °C for 2 hours. After cooling down to room temperature, the mixture was extracted with ether and washed with brine and water. The ether was removed under reduced pressure after drying over $MgSO_4$ and the final product was obtained by distillation. The obtained electrolyte liquids were further dried with calcium hydride and molecular sieves before use.

Molecule **1**, 14,14,15,15,16,16,17,17,18,18,19,19,20,20,21,21,21-heptadecafluoro-2,5,8,11-tetraoxahenicosane. ^{1}H NMR ($CDCl_3$): δ = 2.38-2.48 (m ,2H); 3.39 (s, 3H); 3.55-3.58 (m, 2H); 3.64-3.69 (m, 10H); 3.78 (t, *J* = 7.01 Hz, 2H).

Molecule **2**, 1,1,1,2,2,3,3,4,4-nonafluoro-6-(2-methoxyethoxy)hexane. ^{1}H NMR (500 MHz, $CDCl_3$): δ = 2.40-2.50 (m ,2H); 3.39 (s, 3H); 3.54-3.64 (m, 4H), 3.78 (t, *J* = 7.08 Hz, 2H).

Molecule **3**, 1,1,1,2,2,3,3-heptafluoro-4-(2-methoxyethoxy)butane. ^{1}H NMR (500 MHz, $CDCl_3$): δ = 3.41 (s ,3H); 3.59-3.61 (m, 2H); 3.80-3.81 (m, 2H), 4.05 (t, *J* = 13.98 Hz, 2H).

2. Equations Used in SAXS Model Fitting

Model fitting and size distribution analysis were finally performed by using the Modeling tool from Irena package.[1] The scattering of SAXS measurement employs the polydisperse model which contains the product of form factor and structure factor. Since our electrolyte is dilution system, value of the structure factor could be approximated as 1. For the reason of simplification,

we chose sphere form factor for our the modeling work, assuming the aspect ratio falls in the range of 0.99 and 1.01. Equations involved are listed as follows:

$$I(q) = I_0 V_0^2 \bar{P}(q,r) S_{eff}(q) \tag{1}$$

$$\bar{P}(q,r)\text{^}2 = 3/(qr^3) \times (sin(qr) - (qr \times cos(qr)) \tag{2}$$

$$V_0 = \frac{4}{3} \times pi \times radius\text{^}3 \tag{3}$$

Where $I(q)$ is scattering intensity, q is the scattering vector, V_0 is the volume of the particle, $S_{eff}(q)$ is the effective structure factor, $\bar{P}(q,r)$ is the mean form factor. I_0 is a collection of experimental parameters such as beam intensity, scattering contrast, and experimental set up.

3. Supporting Figures

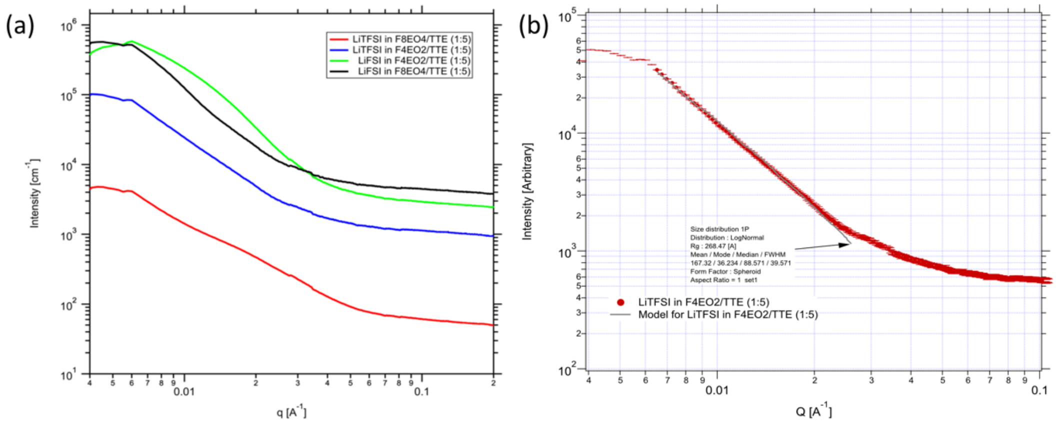

S 1. (a)1D plot of SAXS diffraction patterns for various HFEs solvent electrolyte. (b) Model fitting and size distribution analysis from Irena package for 0.5M LiTFSI in F_4EO_2/TTE 1:5 (v:v) electrolyte.

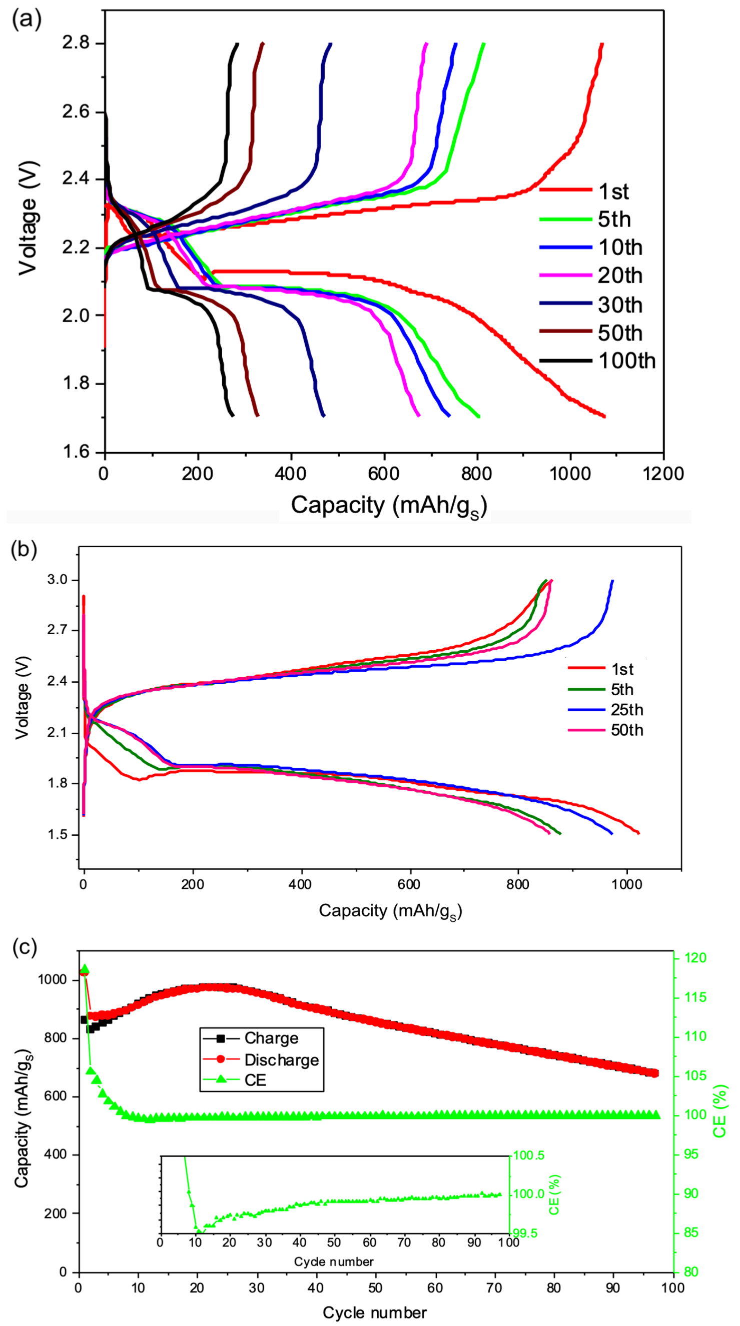

S 2. (a) Voltage profile of baseline electrolyte 1.0M LiTFSI in DME/DOL (V:V=1:1); Electrochemical performance of 0.5M LiTFSI in F_8EO_4/TTE (1:5 volume ratio) electrolyte: (b) voltage profiles and (c) coulombic efficiency and cycling stability.

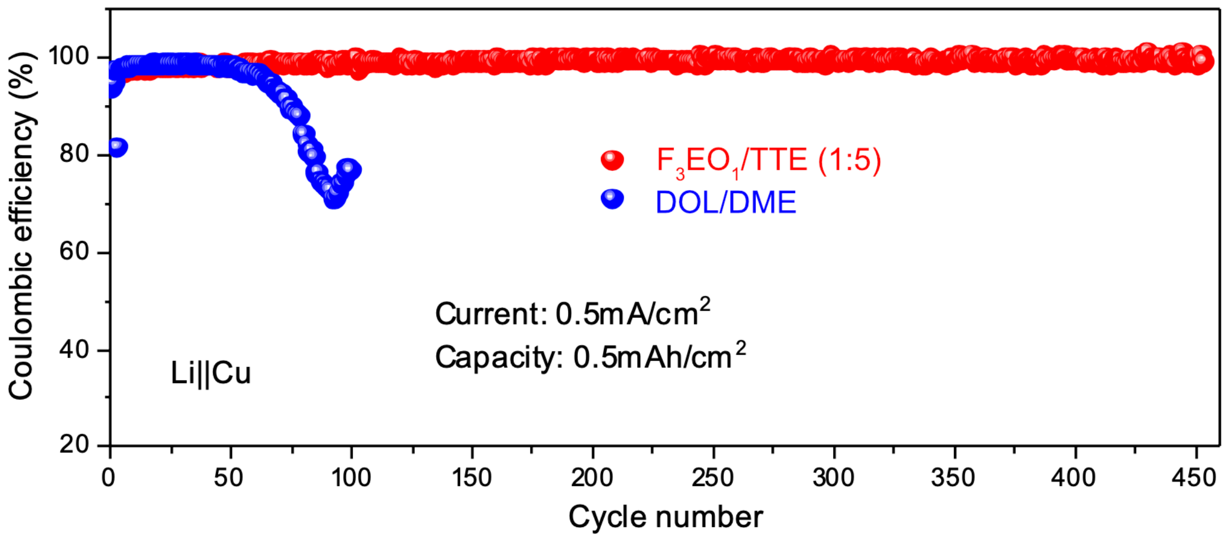

S 3. Coulombic efficiency for 0.5M LiFSI in F_3EO_1/TTE (1:5) electrolyte and 1.0M LiTFSI in DME/DOL (V:V=1:1) electrolyte extracted from Li plating/stripping test at 0.5mA/cm^2 current rate for 1h deposition.

4. Reference

1) Ilavsky, J.; Jemian, P. R., Irena: tool suite for modeling and analysis of small-angle scattering. *Journal of Applied Crystallography* **2009,** *42* (2), 347-353.
